# Supplementary material for: An Italian Single-Center Genomic Surveillance Study: Two-Year Analysis of SARS-CoV-2 Spike Protein Mutations
Source: Int J Mol Sci. 2025 Aug 5;26(15):7558. doi: 10.3390/ijms26157558 (PMC12347092; doi:10.3390/ijms26157558)
Supplement: Supplementary file 1 [file ijms-26-07558-s001.zip › ijms-3752904_Supplementary Material_Round 2.pdf]

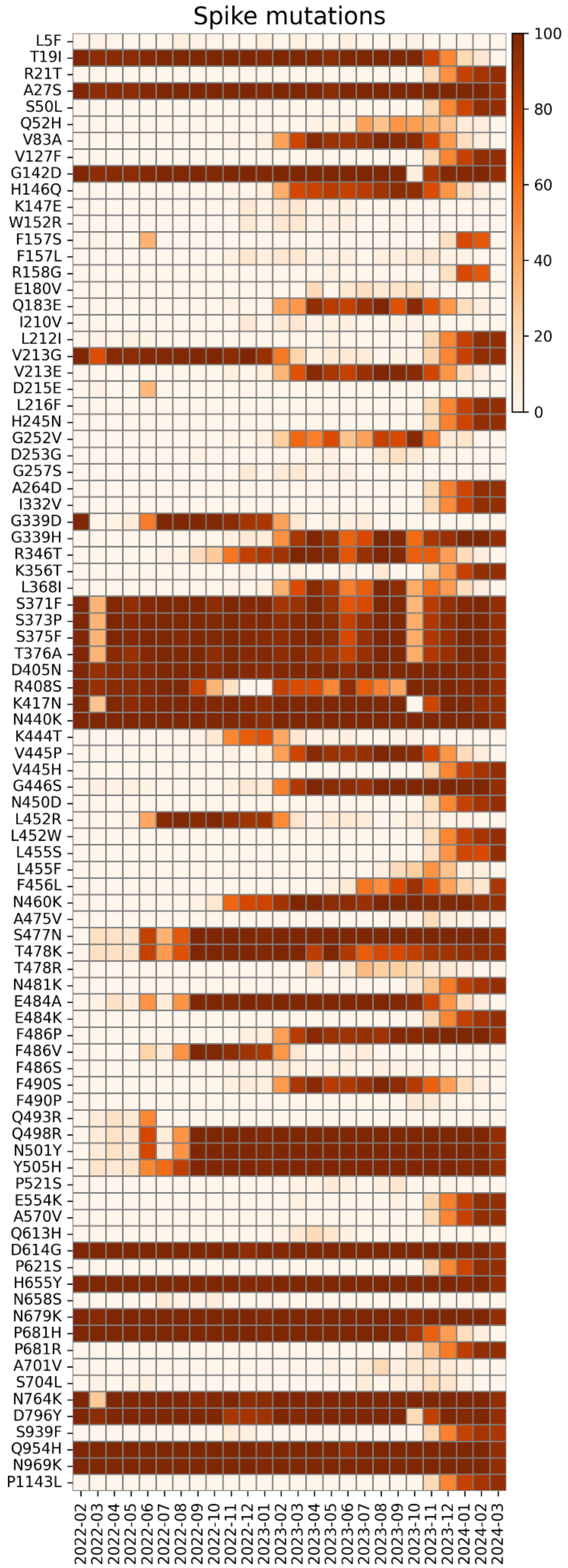

Figure S1. Heatmap of the monthly relative prevalence for all 88 Spike substitutions with total prevalence above 1% in the period from February 2022 to March 2024.

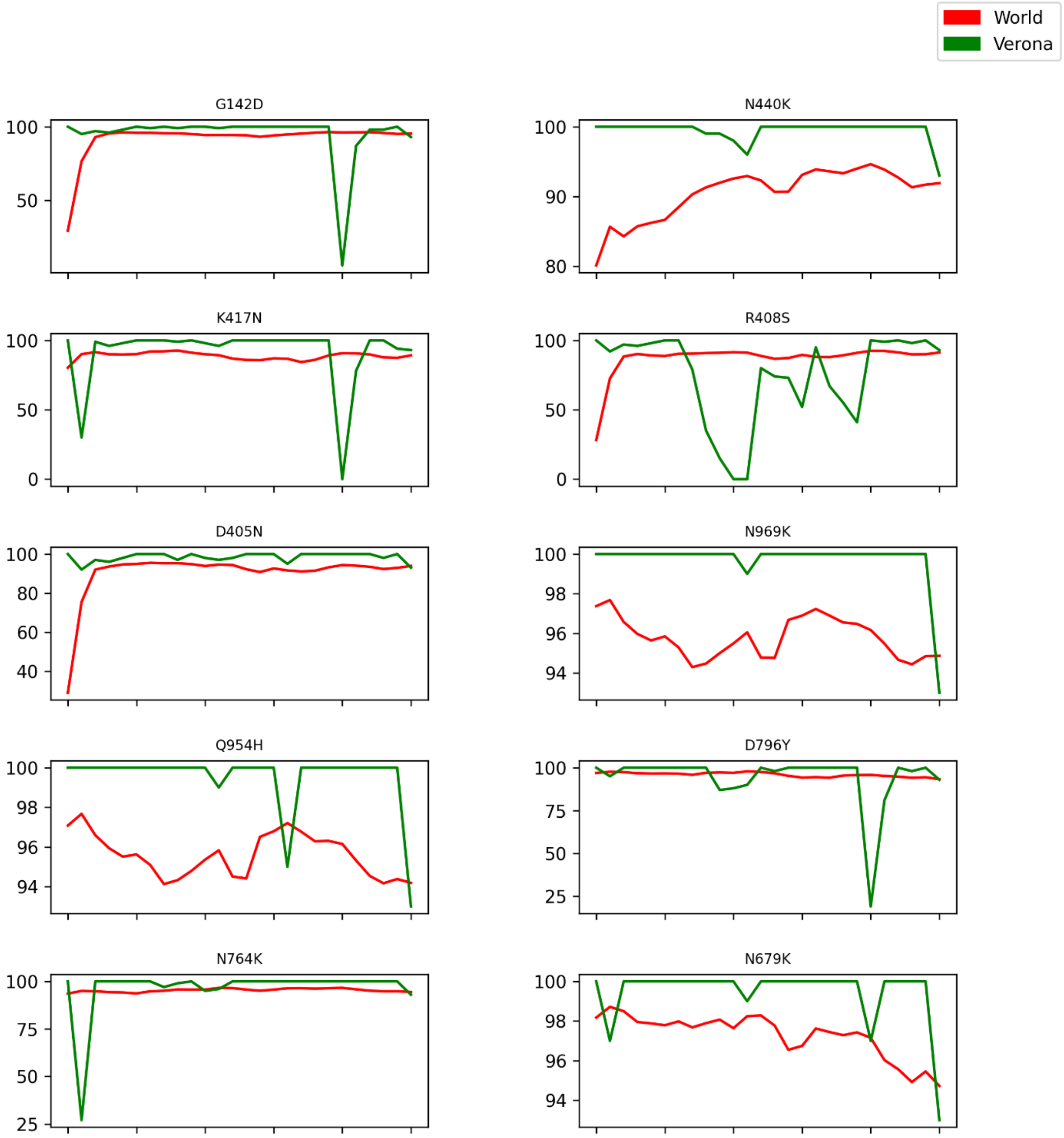

Figure S2. Comparison between relative monthly prevalence in World (red) and Verona (green), for all mutations having a Spearman correlation index below 0.1.

Mutation	Domain	Involvement	Category
A27S	NTD	Immune evasion	Fixed
V127F	NTD	Immune evasion	Emerging
G142D	NTD	Immune evasion and increased viral loads	Fixed
K147E	NTD	Immune evasion	Transient
W152R	NTD	Immune evasion	Transient
F157L	NTD	Immune evasion	Transient
F157S	NTD	Increased transmissibility and reduced ACE2 binding	Divergent
R158G	NTD	Immune evasion and increased viral load	Transient
Q183E	NTD	Immune evasion	Transient
I210V	NTD	Immune evasion	Transient
L212I	NTD	Immune evasion	Divergent
V213G	NTD	Immune evasion and increased infectivity	Divergent
L216F	NTD	Immune evasion	Emerging
H245N	NTD	Immune evasion	Emerging
G252V	NTD	Immune evasion	Transient
D253G	NTD	Immune evasion	Divergent
G257S	NTD	Immune evasion	Transient
A264D	NTD	Immune evasion	Emerging
I332V	RBD	Immune evasion	Emerging
G339H	RBD	Immune evasion	Emerging
G339D	RBD	Immune evasion	Fading
K356T	RBD	Immune evasion and increased infectivity	Divergent
L368I	RBD	Increased ACE2 binding	Transient
S373P	RBD	Increased ACE2 binding	Fixed
D405N	RBD	Immune evasion	Fixed
R408S	RBD	Immune evasion and increased ACE2 binding and	Fixed
K417N	RBD	Immune evasion and reduced binding affinity	Fixed
Q439R	RBD	Increased ACE2 binding	Fading
N440K	RBD	Immune evasion and reduced binding affinity	Fixed
K444T	RBD	Immune evasion	Transient
V445H	RBD	Immune evasion	Emerging
V445P	RBD	Immune evasion	Transient
G446S	RBD	Reduced immune evasion	Divergent
N450D	RBD	Immune evasion	Emerging
L452W	RBD	Immune evasion	Emerging
L452R	RBD	Immune evasion	Transient
L455S	RBD	Immune evasion and reduced ACE2 binding	Emerging
L455F	RBD	Increased ACE2 binding with F456L	Transient
F456L	RBD	Increased ACE2 binding with L455F	Emerging
N460K	RBD	Immune evasion	Emerging
A475V	RBD	Immune evasion	Transient
S477N	RBD	Immune evasion and increased ACE2 binding	Emerging
T478R	RBD	Immune evasion	Transient
T478K	RBD	Increased ACE2 binding	Emerging
N481K	RBD	Immune evasion	Emerging
E484K	RBD	Immune evasion	Emerging
E484A	RBD	Immune evasion and reduced ACE2 binding	Transient

F486P	RBD	Immune evasion	Emerging
F486V	RBD	Immune evasion and reduced ACE2 binding	Transient
F486S	RBD	Immune evasion and reduced ACE2 binding	Transient
F490P	RBD	Immune evasion and reduced ACE2 binding	Transient
F490S	RBD	Immune evasion	Transient
Q498R	RBD	Increased ACE2 binding	Emerging
N501Y	RBD	Immune evasion and increased ACE2 binding	Emerging
Y505H	RBD	Increased ACE2 binding	Emerging
E554K	CTD1	Immune evasion	Emerging
D614G	CTD2	Increased ACE2 binding and increased replication	Fixed
A701V	S2 cleavage site	Reduced ACE2 binding	Transient
D796Y	S2 cleavage site	Immune evasion	Fixed
S939F	HR1	Immune evasion	Divergent
Q954H	HR1	Immune evasion	Fixed

Table S1. List of mutations involved, either positively or negatively, in immune escape and ACE2 binding.
