# Supplementary figures and images for: An Italian Single-Center Genomic Surveillance Study: Two-Year Analysis of SARS-CoV-2 Spike Protein Mutations
Source: Int J Mol Sci. 2025 Aug 5;26(15):7558. doi: 10.3390/ijms26157558 (PMC12347092; doi:10.3390/ijms26157558)

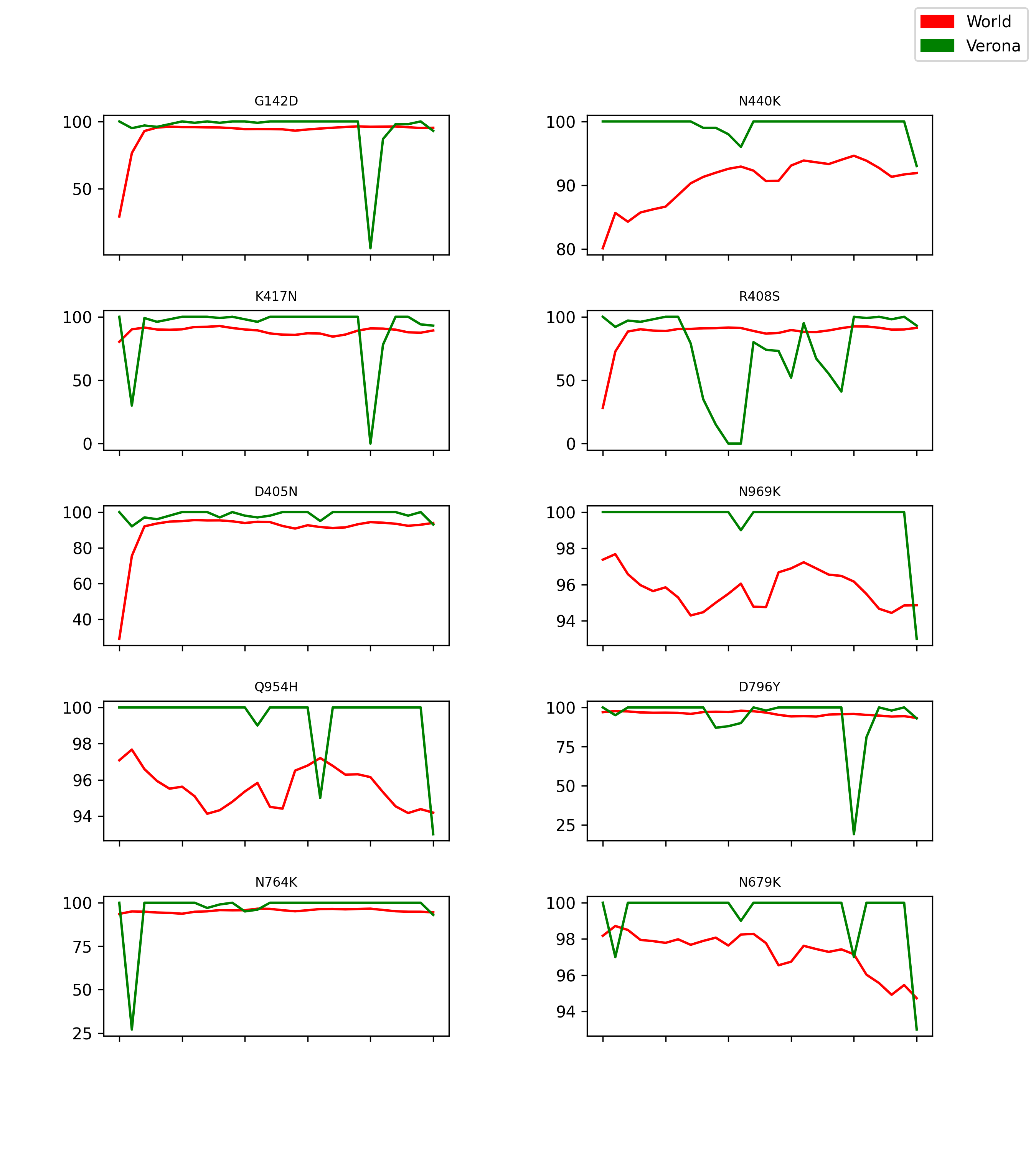

Supplement: Supplementary file 1 [file ijms-26-07558-s001.zip › Figure S2.png]
